# Trends in Out-of-Pocket Costs for and Characteristics of Pharmacy-Dispensed Buprenorphine Medications for Opioid Use Disorder Treatment by Type of Payer, 2015 to 2020

**DOI:** 10.1001/jamanetworkopen.2022.54590

**Published:** 2023-02-10

**Authors:** Andrea E. Strahan, Shaina Desai, Kun Zhang, Gery P. Guy

**Affiliations:** 1 Division of Overdose Prevention, National Center for Injury Prevention and Control, Centers for Disease Control and Prevention, Atlanta, Georgia

## Abstract

**Importance:**

Buprenorphine has been approved for opioid use disorder treatment, yet remains underutilized. Cost may present a barrier; little is known about how out-of-pocket costs vary.

**Objective:**

To determine if out-of-pocket costs and prescription characteristics for buprenorphine varied by type of payer.

**Design, Setting, and Participants:**

This cross-sectional study used all-payer data on retail pharmacy–dispensed buprenorphine prescriptions from January 1, 2015, through December 31, 2020, for adults (aged ≥18 years) in the US, excluding formulations primarily used to treat pain. Data were analyzed from July 2021 to June 2022.

**Exposures:**

Type of payer (private and commercial, self-pay, Medicaid, Medicare, assistance, and unknown) for dispensed prescription.

**Main Outcomes and Measures:**

All outcomes are prescription-level. Mean and median daily out-of-pocket costs were calculated overall and by payer type. Prescription characteristics (days supplied, patient age and sex, generic vs name brand formulations, and prescriber’s location) were examined by payer type.

**Results:**

Although mean daily out-of-pocket costs decreased overall from $4.79 (95% CI, $4.79-$4.80) in 2015 (7 375 508 prescriptions) to $1.91 (95% CI, $1.90-$1.91) in 2020 (13 486 822 prescriptions), out-of-pocket costs continued to vary by payer in 2020. Medicaid had the lowest mean daily out-of-pocket cost across all years—$0.18 (95% CI, $0.18-$0.18) in 2015, and $0.10 (95% CI, $0.10-$0.10) in 2020. Private and commercial paid prescriptions fell from $4.80 (95% CI, $4.79-$4.81) per day in 2015 to $1.82 (95% CI, $1.82-$1.83) in 2020. Self-pay and assistance categories had the highest mean daily out-of-pocket costs across study years ($9.76 [95% CI, $9.74-$9.78] and $8.72 [95% CI, $8.71-$8.73], respectively, in 2015; $8.44 [95% CI, $8.43-$8.46] and $6.31 [95% CI, $6.30-$6.31], respectively, in 2020). Medicaid paid prescriptions had a mean supply of 15.59 days (95% CI, 15.58-15.59 days) and the lowest percentage of generic prescriptions (57.88%; 95% CI, 57.84%-57.92%). Out-of-pocket cost varied by prescriber location and patient characteristics; mean costs were highest for prescriptions written in the South ($2.91; 95% CI, $2.90-$2.91), metropolitan counties ($1.93; 95% CI, $1.93-$1.93), and for individuals aged 35 to 44 years ($2.10; 95% CI, $2.09-$2.10).

**Conclusions and Relevance:**

This cross-sectional study found that mean daily out-of-pocket costs for buprenorphine were lower in 2020 than in 2015, but variation by payer existed in all study years. Financial barriers to accessing and maintaining buprenorphine for opioid use disorder treatment may exist and differ by type of prescription coverage. Future research could monitor costs and identify potential barriers that may impact access and retention in care.

## Key Points

**Question:**

How do out-of-pocket costs and prescription characteristics for buprenorphine prescriptions approved for opioid use disorder treatment vary by payer?

**Findings:**

Cross-sectional analysis was used to calculate daily out-of-pocket costs and prescription characteristics for retail pharmacy–dispensed buprenorphine from 2015 to 2020. Mean daily out-of-pocket costs decreased overall from $4.79 in 2015 (7 375 508 prescriptions) to $1.91 in 2020 (13 486 822 prescriptions), but out-of-pocket costs continued to vary by payer in 2020.

**Meaning:**

These findings suggest that financial barriers to accessing and maintaining buprenorphine treatment for opioid use disorder may exist and differ by type of and access to prescription coverage.

## Teaser

This cross-sectional study evaluates trends in out-of-pocket costs for and characteristics of pharmacy-dispensed buprenorphine medications for opioid use disorder treatment by type of payer from 2015 to 2020.

## Introduction

In 2020, approximately 68 630 opioid-involved drug overdose deaths occurred and 2.7 million people had opioid use disorder (OUD) in the US.^1,2^ Buprenorphine is a US Food and Drug Administration–approved medication for opioid use disorder (MOUD) that can be prescribed in any medical setting by waivered clinicians.^3^ Longer buprenorphine retention and better adherence are associated with less opioid use,^4,5^ and buprenorphine is associated with a 50% decrease in mortality risk.^6^ Improving access to evidence-based MOUD treatment is an important component of overdose prevention,^7^ and new telehealth flexibilities were introduced in 2020 for waivered clinicians in response to the COVID-19 pandemic.^8^

Buprenorphine remains underutilized, possibly due to barriers including clinician availability, wait times, and cost.^3^ In 2020, only 11.2% of individuals with OUD reported receiving MOUD.^2^ Higher buprenorphine out-of-pocket costs (costs not reimbursed by insurance) were associated with lower treatment retention and adherence among a commercially insured population.^9^ Prior research found that median out-of-pocket costs for a 30-day buprenorphine prescription decreased from $67 to $32 between 2003 and 2015 among a commercially insured population.^10^ Little is known about out-of-pocket costs in more recent years or how these costs vary by payer. This study examines how daily out-of-pocket costs and characteristics of buprenorphine MOUD prescriptions dispensed via retail pharmacies varied by payer from 2015 through 2020.

## Methods

Data are from the IQVIA Longitudinal Prescription (LRx) database, capturing 92% of retail pharmacy prescriptions in the US. We followed the Strengthening the Reporting of Observational Studies in Epidemiology (STROBE) reporting guideline reporting guideline. The US Centers for Disease Control and Prevention determined human participant regulations, including informed participant consent, and institutional review board review was not applicable because deidentified secondary data were used in accordance with 45 CFR §46.

Buprenorphine prescriptions dispensed to adults (aged ≥18 years) from January 1, 2015, through December 31, 2020, were identified. Buprenorphine formulations primarily used to treat pain (Butrans and Belbuca) were not included in the sample. Prescriptions missing cost (2 917 421 prescriptions) and approximately 0.1% of the sample with negative or exceedingly high cost values (67 631 prescriptions) were excluded. The final sample was 67 592 683 prescriptions from 2015 to 2020.

Prescription payers captured in the LRx data were combined into 6 categories: private and commercial, self-pay, Medicaid, Medicare, assistance, and unknown. Private and commercial included several forms of employer-sponsored health insurance, plans purchased through health insurance exchanges, and those administered by pharmacy benefit managers. Self-pay indicates a prescription was paid for entirely with cash. Medicaid includes Medicaid managed care or fee-for-service Medicaid. Medicare indicates prescriptions paid by Medicare Part D. The assistance category indicates payment using a discount card (including non-Medicare senior discount cards), a coupon, or a voucher. The unknown category was composed of prescriptions that were missing payer type or had “unspecified third party” or “unknown” for payer type (more detail on payer categories is provided in eTable 1 in Supplement 1).

### Statistical Analysis

Analysis was cross-sectional and all outcomes were prescription-level. Daily out-of-pocket costs were calculated to facilitate comparisons across prescriptions of differing duration. This analysis examined daily out-of-pocket costs by calculating mean costs with 95% CIs and median costs with IQRs, overall and by payer type. Mean days supply with 95% CI and percentage of generic (vs name brand) prescriptions with 95% CI were also calculated. Mean daily out-of-pocket costs by prescription characteristics were examined, including age and sex of patient in receipt of the prescription and US census region^11^ and metropolitan/nonmetropolitan status^12^ (both according to the county where the prescriber was professionally located). Nonmetropolitan counties were those designated as micropolitan statistical areas or noncore counties. Costs were adjusted to 2020 dollars using the Average Consumer Price Index Research Series for 2020.^13^ This descriptive study did not involve statistical tests. Stata version 14.2 (StataCorp) was used for all analyses. The analysis was conducted from July 2021 to June 2022.

## Results

There were 7 375 508 buprenorphine MOUD prescriptions dispensed in 2015 and 13 486 822 in 2020 (Table 1). In 2015, private and commercial paid for 26.99% (95% CI, 26.96%-27.02%) of prescriptions (1 990 569 prescriptions) and Medicaid paid for 23.55% (95% CI, 23.52%-23.58%) of prescriptions (1 736 811 prescriptions); in 2020, private and commercial paid for 20.57% (95% CI, 20.55%-20.59%; 2 774 396 prescriptions), while Medicaid paid for 43.00% (95% CI, 42.98%-43.03%; 5 799 935 prescriptions). In 2015, Medicare accounted for the smallest percentage, 5.91% (95% CI, 5.90%-5.93%; 436 118 prescriptions), but increased to 10.16% (95% CI, 10.15%-10.18%; 1 370 901 prescriptions) in 2020. In 2015, assistance accounted for 22.96% (95% CI, 22.93%-22.99%) of prescriptions (1 693 406 prescriptions) and self-pay 11.52% (95% CI, 11.50%-11.55%; 850 021 prescriptions); in 2020, self-pay was 6.39% (95% CI, 6.38%-6.40%; 861 977 prescriptions), while assistance accounted for 12.17% (95% CI, 12.16%-12.19%; 1 641 711 prescriptions).

**Table 1.** Number of Retail Pharmacy–Dispensed Buprenorphine Prescriptions and Mean and Median Daily Out-of-Pocket Cost by Type of Payer, 2015 to 2020^a^

	Overall	Payer					
		Private and commercial	Self-pay	Medicaid	Medicare	Assistance	Unknown
2015 Prescriptions							
No.	7 375 508	1 990 569	850 021	1 736 811	436 118	1 693 406	668 583
% (95% CI)		26.99 (26.96-27.02)	11.52 (11.50-11.55)	23.55 (23.52-23.58)	5.91 (5.90-5.93)	22.96 (22.93-22.99)	9.06 (9.04-9.09)
Median (IQR), $	0.73 (0.00-8.49)	1.56 (0.36-7.24)	8.98 (0.00-15.02)	0.04 (0.00-0.22)	0.13 (0.00-0.39)	8.67 (0.73-12.07)	1.21 (0.31-3.12)
Mean (95% CI), $	4.79 (4.79-4.80)	4.80 (4.79-4.81)	9.76 (9.74-9.78)	0.18 (0.18-0.18)	0.98 (0.97-0.98)	8.72 (8.71-8.73)	3.02 (3.01-3.03)
2016 Prescriptions							
No.	9 895 979	2 485 602	1 050 754	2 685 588	645 997	2 144 935	883 103
% (95% CI)		25.12 (25.09-25.14)	10.62 (10.60-10.64)	27.14 (27.11-27.17)	6.53 (6.51-6.54)	21.67 (21.65-21.70)	8.92 (8.91-8.94)
Median (IQR), $	0.65 (0.00-8.09)	1.35 (0.22-5.39)	9.58 (2.40-16.46)	0.00 (0.00-0.20)	0.13 (0.04-0.36)	10.05 (4.93-13.88)	1.26 (0.32-3.08)
Mean (95% CI), $	4.74 (4.74-4.75)	4.11 (4.10-4.12)	10.60 (10.58-10.62)	0.17 (0.16-0.17)	0.93 (0.93-0.94)	10.23 (10.22-10.24)	2.95 (2.94-2.96)
2017 Prescriptions							
No.	11 183 369	2 557 630	1 066 366	3 562 623	814 353	2 224 492	957 905
% (95% CI)		22.87 (22.85-22.89)	9.54 (9.52—9.55)	31.86 (31.83-31.88)	7.28 (7.27-7.30)	19.89 (19.87-19.91)	8.57 (8.55-8.58)
Median (IQR), $	0.45 (0.00-6.82)	1.23 (0.18-4.05)	9.50 (3.63-15.31)	0.00 (0.00-0.15)	0.13 (0.04-0.35)	9.98 (5.32-13.97)	1.23 (0.35-2.84)
Mean (95% CI), $	4.21 (4.21-4.21)	3.51 (3.51-3.52)	10.34 (10.33-10.36)	0.15 (0.15-0.15)	0.90 (0.90-0.91)	10.41 (10.40-10.42)	2.77 (2.76-2.78)
2018 Prescriptions							
No.	12 407 065	2 753 107	1 114 372	4 472 262	1 019 243	2 096 230	951 851
% (95% CI)		22.19 (22.17-22.21)	8.98 (8.97-9.00)	36.05 (36.02-36.07)	8.22 (8.20-8.23)	16.90 (16.87-16.92)	7.67 (7.66-7.69)
Median (IQR), $	0.34 (0.00-5.02)	1.20 (0.23-3.97)	8.76 (3.68-13.57)	0.00 (0.00-0.13)	0.13 (0.04-0.41)	8.40 (4.66-14.52)	1.37 (0.39-2.95)
Mean (95% CI), $	3.60 (3.60-3.61)	3.44 (3.44-3.45)	9.64 (9.63-9.66)	0.13 (0.13-0.13)	0.84 (0.84-0.85)	9.71 (9.70-9.72)	2.84 (2.83-2.85)
2019 Prescriptions							
No.	13 243 940	2 940 798	1 083 706	5 208 829	1 236 955	1 733 993	1 039 659
% (95% CI)		22.20 (22.18-22.23)	8.18 (8.17-8.20)	39.33 (39.30-39.36)	9.34 (9.32-9.36)	13.09 (13.07-13.11)	7.85 (7.84-7.86)
Median (IQR), $	0.18 (0.00-2.74)	0.67 (0.11-2.53)	8.10 (4.05-12.15)	0.00 (0.00-0.11)	0.11 (0.04-0.29)	6.47 (3.87-10.84)	0.84 (0.34-2.36)
Mean (95% CI), $	2.60 (2.60-2.60)	2.43 (2.43-2.44)	9.00 (8.99-9.02)	0.11 (0.11-0.11)	0.62 (0.61-0.62)	7.95 (7.94-7.96)	2.33 (2.32-2.34)
2020 Prescriptions							
No.	13 486 822	2 774 396	861 977	5 799 935	1 370 901	1 641 711	1 037 902
% (95% CI)		20.57 (20.55-20.59)	6.39 (6.38-6.40)	43.00 (42.98-43.03)	10.16 (10.15-10.18)	12.17 (12.16-12.19)	7.70 (7.68-7.71)
Median (IQR), $	0.12 (0.00-1.48)	0.50 (0.17-1.67)	7.50 (4.00-11.71)	0.00 (0.00-0.04)	0.12 (0.04-0.29)	5.49 (3.18-7.82)	0.65 (0.33-1.67)
Mean (95% CI), $	1.91 (1.90-1.91)	1.82 (1.82-1.83)	8.44 (8.43-8.46)	0.10 (0.10-0.10)	0.46 (0.46-0.46)	6.31 (6.30-6.31)	1.73 (1.72-1.74)

^a^ Authors’ analysis of IQVIA Longitudinal Prescription (LRx) database from 2015 to 2020. Sample includes buprenorphine prescriptions dispensed to adults (aged ≥18 years old) in the US. Buprenorphine formulations (Butrans and Belbuca) primarily used to treat pain were excluded. All percentages are row percentages. Private and commercial included several forms of employer-sponsored health insurance, plans purchased through health insurance exchanges, and those administered by pharmacy benefit managers. Self-pay indicates a prescription was paid for entirely with cash. Medicaid includes Medicaid managed care or fee-for-service Medicaid. Medicare indicates prescriptions paid by Medicare Part D. The assistance category indicates payment using a discount card (including non-Medicare senior discount cards), a coupon, or a voucher. The unknown category was composed of prescriptions that were missing payer type or had “unspecified third party” or “unknown” for payer type.

Overall mean daily out-of-pocket costs decreased from $4.79 (95% CI, $4.79-$4.80) in 2015 to $1.91 (95% CI, $1.90-$1.91) in 2020. Medicaid had the lowest mean out-of-pocket cost in all years—$0.18 (95% CI, $0.18-$0.18) in 2015, $0.10 (95% CI, $0.10-$0.10) in 2020—and $0.00 median out-of-pocket cost from 2016 to 2020. Private and commercial mean cost fell from $4.80 (95% CI, $4.79-$4.81) in 2015 to $1.82 (95% CI, $1.82-$1.83) in 2020, but was still higher than Medicaid ($0.10; 95% CI, $0.10-$0.10) or Medicare ($0.46; 95% CI, $0.46-$0.46) in 2020. Self-pay and assistance had the highest mean out-of-pocket costs; self-pay was $9.76 (95% CI, $9.74-$9.78) in 2015, $10.60 (95% CI, $10.58-$10.62) in 2016, and $8.44 (95% CI, $8.43-$8.46) in 2020; the assistance category was $8.72 (95% CI, $8.71-$8.73) in 2015, $10.41 (95% CI, $10.40-$10.42) in 2017, and $6.31 (95% CI, $6.30-$6.31) in 2020.

Among prescriptions dispensed in 2020, private and commercial (21.37 days; 95% CI, 21.36-21.38 days), Medicare (20.60 days; 95% CI, 20.59-20.62 days), and unknown (22.63 days; 95% CI, 22.61-22.65 days) had longer mean durations, while those paid by self-pay had the shortest (12.62 days; 95% CI, 12.60-12.65 days) (Table 2). Medicaid paid prescriptions had a mean days supply of 15.59 days (95% CI, 15.58-15.59 days). Generic prescriptions accounted for 94.81% (95% CI, 94.77%-94.86%) of self-pay vs 57.88% (95% CI, 57.84%-57.92%) of Medicaid. The lower percentage of Medicaid-paid generic prescriptions was associated with a higher proportion of Suboxone prescriptions (40.33%) than other payers (eTable 2 in Supplement 1).

**Table 2.** Mean Days Supplied, Generic Status, and Daily Out-of-Pocket Cost by Clinician Location and Patient Characteristics for Retail Pharmacy–Dispensed Buprenorphine Prescriptions by Type of Payer, 2020^a^

Characteristic	Primary insurance payer						
	Overall	Private and commercial	Self-pay	Medicaid	Medicare	Assistance	Unknown
No.	13 486 822	2 774 396	861 977	5 799 935	1 370 901	1 641 711	1 037 902
Days supplied, mean (95% CI)	17.59 (17.59-17.60)	21.37 (21.36-21.38)	12.62 (12.60-12.65)	15.59 (15.58-15.59)	20.60 (20.59-20.62)	15.22 (15.21-15.24)	22.63 (22.61-22.65)
Generic, % (95% CI)^b^	74.37(74.34-74.39)	88.16 (88.13-88.20)	94.81(94.77-94.86)	57.88(57.84-57.92)	76.54(76.47-76.61)	88.93(88.88-88.98)	86.70(86.64-86.77)
**Out-of-pocket cost by clinician location and patient characteristics, mean (95% CI), $ [No. prescriptions]**							
US census region^b^							
Northeast	1.04 (1.04-1.04) [3 495 861]	1.13 (1.13-1.14) [757 002]	8.02 (8.00-8.06) [125 778]	0.12 (0.12-0.12) [1 788 530]	0.36 (0.35-0.36) [388 878]	5.26 (5.24-5.28) [209 924]	1.40 (1.39-1.41) [225 749]
Midwest	1.32 (1.32-1.32) [2 661 858]	1.62 (1.62-1.63) [546 463]	7.53 (7.48-7.57) [87 014]	0.05 (0.05-0.05) [1 358 633]	0.44 (0.44-0.45) [250 412]	5.51 (5.50-5.53) [263 416]	2.18 (2.16-2.20) [155 920]
South	2.91 (2.90-2.91) [5 654 494]	2.65 (2.65-2.66) [1 070 088]	8.73 (8.72-8.75) [587 623]	0.12 (0.12-0.12) [1 942 310]	0.52 (0.51-0.52) [537 318]	6.94 (6.93-6.95) [1 011 868]	1.85 (1.84-1.86) [505 287]
West	1.27 (1.26-1.27) [1 674 609]	1.19 (1.18-1.20) [400 843]	7.84 (7.79-7.90) [61 562]	0.11 (0.11-0.12) [710 462]	0.53 (0.53-0.54) [194 293]	4.95 (4.93-4.97) [156 503]	1.36 (1.35-1.38) [150 946]
Urbanization status^c^							
Metropolitan	1.93 (1.93-1.93) [11 203 064]	1.76 (1.76-1.77) [2 401 739]	8.44 (8.43-8.46) [708 505]	0.10 (0.10-0.10) [4 625 500]	0.47 (0.47-0.47) [1 142 311]	6.27 (6.27-6.28) [1 408 458]	1.71 (1.71-1.72) [916 551]
Nonmetropolitan	1.80 (1.79-1.80) [2 283 758]	2.22 (2.21-2.24) [372 657]	8.44 (8.41-8.47) [153 472]	0.12 (0.12-0.12) [1 174 435]	0.43 (0.42-0.43) [228 590]	6.50 (6.48-6.52) [233 253]	1.88 (1.86-1.90) [121 351]
Patient age							
18-24	1.46 (1.45-1.48) [326 955]	1.55 (1.53-1.57) [83 670]	7.78 (7.69-7.87) [17 754]	0.08 (0.08-0.08) [166 429]	0.41 (0.37-0.45) [4752]	5.70 (5.65-5.75) [26 176]	1.65 (1.61-1.68) [28 174]
25-34	1.92 (1.92-1.92) [3 952 591]	1.99 (1.99-2.00) [705 577]	8.12 (8.10-8.15) [285 989]	0.10 (0.10-0.10) [2 084 809]	0.27 (0.26-0.28) [85 015]	6.07 (6.06-6.09) [516 335]	1.77 (1.76-1.78) [274 866]
35-44	2.10 (2.09-2.10) [4 749 614]	1.96 (1.95-1.97) [999 078]	8.63 (8.61-8.65) [330 205]	0.11 (0.10-0.11) [2 128 248]	0.27 (0.27-0.28) [258 785]	6.47 (6.45-6.48) [640 887]	1.81 (1.80-1.82) [392 411]
45-54	1.94 (1.94-1.95) [2 388 516]	1.75 (1.74-1.76) [537 370]	8.81 (8.77-8.84) [142 735]	0.10 (0.09-0.10) [911 736]	0.35 (0.34-0.35) [300 830]	6.54 (6.52-6.56) [291 143]	1.67 (1.66-1.68) [204 702]
55-64	1.53 (1.53-1.54) [1 519 464]	1.46 (1.45-1.47) [351 239]	8.36 (8.31-8.41) [67 867]	0.10 (0.10-0.11) [455 179]	0.48 (0.48-0.49) [395 291]	6.29 (6.27-6.32) [131 842]	1.52 (1.51-1.54) [118 046]
≥65	1.32 (1.31-1.33) [549 682]	1.14 (1.13-1.16) [97 462]	8.21 (8.11-8.31) [17 427]	0.12 (0.11-0.13) [53 534]	0.74 (0.73-0.74) [326 228]	5.42 (5.37-5.47) [35 328]	1.63 (1.59-1.67) [19 703]
Patient sex							
Male	2.11 (2.11-2.11) [7 348 155]	1.83 (1.83-1.84) [1 729 007]	8.40 (8.38-8.41) [490 489]	0.11 (0.11-0.11) [2 739 590]	0.54 (0.54-0.54) [701 586]	6.31 (6.30-6.32) [1 006 889]	1.74 (1.74-1.75) [680 594]
Female	1.66 (1.66-1.67) [6 136 395]	1.81 (1.81-1.82) [1 045 225]	8.51 (8.49-8.53) [370 754]	0.10 (0.10-0.10) [3 059 382]	0.38 (0.38-0.38) [669 156]	6.30 (6.29-6.32) [634 779]	1.71 (1.70-1.72) [357 099]
Unknown	3.01 (2.77-3.25) [2272]	2.06 (1.63-2.49) [164]	6.28 (5.69-6.88) [734]	0.10 (0.05-0.15) [963]	0.23 (0.11-0.35) [159]	4.65 (4.19-5.11) [43]	7.41 (6.78-8.05) [209]

^a^ Authors’ analysis of IQVIA Longitudinal Prescription (LRx) database in 2020. Sample includes buprenorphine prescriptions dispensed to adults (≥18 years old) in the US. Buprenorphine formulations (Butrans and Belbuca) primarily used to treat pain were excluded. Private and commercial included several forms of employer-sponsored health insurance, plans purchased through health insurance exchanges, and those administered by pharmacy benefit managers. Self-pay indicates a prescription was paid for entirely with cash. Medicaid includes Medicaid managed care or fee-for-service Medicaid. Medicare indicates prescriptions paid by Medicare Part D. The assistance category indicates payment using a discount card (including non-Medicare senior discount cards), a coupon, or a voucher. The unknown category was composed of prescriptions that were missing payer type or had “unspecified third party” or “unknown” for payer type.

^b^ A total of 615 observations were missing information on product name/generic status (120 private and commercial; 56 self-pay; 186 Medicaid; 175 Medicare; 1 assistance; 75 unknown).

^c^ According to the county where the prescriber was professionally located.

Across payers, daily mean out-of-pocket costs were highest in the South at $2.91 (95% CI, $2.90-$2.91) and lowest in the Northeast at $1.04 (95% CI, $1.04-$1.04) (Table 2). More than half of prescriptions in self-pay (587 623 prescriptions) and assistance categories (1 011 868 prescriptions) were prescribed in the South. Among private and commercial, Medicaid, and Medicare, the highest number of prescriptions (1 070 088, 1 942 310, and 537 318 prescriptions, respectively) were prescribed in the South, followed by the Northeast (757 002, 1 788 530, and 388 878 prescriptions, respectively). Overall and across all payer categories, the highest number of prescriptions were prescribed in metropolitan counties. Mean daily out-of-pocket cost was highest in metropolitan counties at $1.93 (95% CI, $1.93-$1.93), vs a mean cost of $1.80 (95% CI, $1.79-$1.80) in nonmetropolitan counties. Similarly (with exception of Medicare-paid prescriptions), the highest number of prescriptions for each type of payer were dispensed to individuals aged 35 to 44 years and overall mean daily out-of-pocket cost was highest for this group at $2.10 (95% CI, $2.09-$2.10). A higher number of prescriptions were dispensed to males than females in each payer category, except Medicaid.

## Discussion

This study found that out-of-pocket costs for buprenorphine prescriptions have decreased since 2015 and costs continued to vary by payer in 2020. In 2020, an individual would face daily mean out-of-pocket costs of $0.10 for a prescription paid by Medicaid or $0.46 for one paid by Medicare, versus $1.82 for a prescription paid by private and commercial. Furthermore, for the approximately 1-in-5 prescriptions dispensed in 2020 that were paid by assistance or self-pay, mean daily out-of-pocket costs were $6.31 or $8.44, respectively. These daily costs can add to up substantial costs for patients retained on long-term therapy or receiving longer duration prescriptions, such as for a 30-days supply. Variation in mean out-of-pocket costs also exists by clinician location and patient characteristics, suggesting that financial barriers for individuals accessing and maintaining MOUD treatment may depend on how one pays for prescriptions and factors influencing access to prescription coverage.

Medicaid paid for the largest number of prescriptions and increased most from 2015 to 2020. Prescriptions covered by Medicaid differed from other payers, with a shorter mean duration and a greater proportion of nongeneric prescriptions, potentially due to state Medicaid requirements that afford preferred status to certain forms of buprenorphine for Medicaid-covered prescriptions.^14^ Future studies might examine the reason for the shorter duration in the Medicaid category and its potential implication for retention in MOUD treatment, as well as if there are differences in outcomes for patients using different types of buprenorphine, including generic vs name-brand buprenorphine formulations.

Study findings on daily out-of-pocket costs for private and commercial paid prescriptions are consistent with prior research. Dunphy et al^9^ used data on individuals with insurance coverage from large self-insured employers and found that among those retained on buprenorphine treatment for less than 180 days, the 2016 to 2017 mean daily cost was $2.63. Dunphy et al also found a $1 increase in daily out-of-pocket buprenorphine cost was associated with a 12–14% decrease in the odds of MOUD treatment retention, highlighting the importance of this study’s findings. Greater financial barriers to treatment retention might exist according to payer, particularly for self-pay patients and those using assistance.

### Limitations

Study limitations include that the IQVIA data do not allow differentiation between prescriptions received for MOUD vs those diverted to other uses, such as off-label use for pain. The IQVIA LRx data are not weighted to be geographically representative. Prescription coverage may be lower in certain regions, such as those with higher numbers of health maintenance organizations, which may dispense prescriptions through their own pharmacies. Information on patient race and ethnicity was not available in the data. Any inaccuracies in clinician address have the potential to bias estimates at the county level. Additionally, this analysis only included retail pharmacy–dispensed buprenorphine and did not examine other MOUD types, such as methadone or extended-release naltrexone.

## Conclusions

In this cross-sectional study of all-payer pharmaceutical data, findings showed that while mean daily out-of-pocket costs for buprenorphine decreased between 2015 and 2020, costs continued to vary by payer and remain high for some in 2020. Public health and insurer strategies aimed at reducing higher patient out-of-pocket costs may address financial barriers and improve buprenorphine treatment retention. Future research might continue to examine how cost-sharing may impact MOUD treatment initiation and retention under different payers.

## Notes

Supplement 1. **eTable 1.** Payer Categories **eTable 2. **Product Name Among Retail Pharmacy–Dispensed Buprenorphine Prescriptions by Payer Type, 2020

Supplement 2.
Data Sharing Statement
