# Supplementary material for: Trends in Out-of-Pocket Costs for and Characteristics of Pharmacy-Dispensed Buprenorphine Medications for Opioid Use Disorder Treatment by Type of Payer, 2015 to 2020
Source: JAMA Netw Open. 2023 Feb 10;6(2):e2254590. doi: 10.1001/jamanetworkopen.2022.54590 (PMC9918874; doi:10.1001/jamanetworkopen.2022.54590)
Supplement: Supplement 1. — eTable 1. Payer Categories eTable 2. Product Name Among Retail Pharmacy–Dispensed Buprenorphine Prescriptions by Payer Type, 2020 [file jamanetwopen-e2254590-s001.pdf]

Supplemental Online Content

Strahan AE, Desai S, Zhang K, Guy GP Jr. Trends in out-of-pocket costs for and characteristics of pharmacy-dispensed buprenorphine medications for opioid use disorder treatment by type of payer, 2015 to 2020. *JAMA Netw Open*. 2023;6(2):e2254590. doi:10.1001/jamanetworkopen.2022.54590

eTable 1. Payer Categories

eTable 2. Product Name Among Retail Pharmacy–Dispensed Buprenorphine Prescriptions by Payer Type, 2020

This supplemental material has been provided by the authors to give readers additional information about their work.

© 2023 Strahan AE et al. *JAMA Network Open.*

eTable 1. Payer Categories

Prescription payers captured in the LRx data were combined into six payer categories: private/commercial, self-pay, Medicaid, Medicare, assistance, unknown. Private/commercial included several forms of employer-sponsored health insurance, plans purchased through health insurance exchanges, and those administered by pharmacy benefit managers. Self-pay indicates a prescription was paid for entirely with cash. Medicaid includes Medicaid managed care or fee-for-service Medicaid. Medicare indicates prescriptions paid by Medicare Part D. The assistance category indicates payment using a discount card (including non-Medicare senior discount cards), a coupon, or a voucher. The unknown category was comprised of prescriptions that were missing payer type or had “unspecified third party” or “unknown” for payer type.

Payer Category	Prescription Payer
Private/Commercial	Behavioral Health
Private/Commercial	Consumer Directed Health Plan
Private/Commercial	HMO - Combination Model
Private/Commercial	Employer-Sponsored CMS Retiree Prescription Drug Program
Private/Commercial	Employer-Sponsored PBM Retiree Prescription Drug Program
Private/Commercial	Employer
Private/Commercial	Exclusive Provider Organization
Private/Commercial	Federal Assistance Program
Private/Commercial	Federal Employee
Private/Commercial	HMO - Group Practice Model
Private/Commercial	Health Insurance Exchange EPO Plan
Private/Commercial	Health Insurance Exchange EPO General
Private/Commercial	Health Insurance Exchange General
Private/Commercial	Health Insurance Exchange HMO
Private/Commercial	Health Insurance Exchange HMO General
Private/Commercial	Health Insurance Exchange POS Plan
Private/Commercial	Health Insurance Exchange POS General
Private/Commercial	Health Insurance Exchange PPO
Private/Commercial	Health Insurance Exchange PPO General
Private/Commercial	HMO
Private/Commercial	HMO - Independent Practice Association Model
Private/Commercial	HMO - Network Model
Private/Commercial	Non-HMO
Private/Commercial	Pharmacy Benefit Manager
Private/Commercial	PBM Book of Business - Unidentified Plans
Private/Commercial	Point of Service
Private/Commercial	Preferred Provider Organization
Private/Commercial	Claims Processor
Private/Commercial	HMO - Staff Model
Private/Commercial	State Assistance Program
Private/Commercial	State Employees
Private/Commercial	Third Party Administrator
Private/Commercial	Union
Private/Commercial	Worker's Compensation
Self-Pay	Cash
Medicaid	Children's Health Insurance Program
Medicaid	Fee-for-Service Medicaid
Medicaid	Managed Medicaid

© 2023 Strahan AE et al. *JAMA Network Open.*

Medicaid	Managed Medicaid-includes some other type of membership such as state assistance
Medicare	Dual Eligible Medicare Medicaid Plan
Medicare	Medicare Discount Card Program
Medicare	Medicare A
Medicare	Medicare D Advantage - Plan Specific
Medicare	General Medicare D Advantage
Medicare	Medicare B
Medicare	Medicare D Prescription Drug Program - Plan Specific
Medicare	General Medicare D Prescription Drug Program
Medicare	Medicare D Special Needs Plan
Medicare	General Medicare D Special Needs Plan
Medicare	Medicare D Unspecified
Medicare	Medicare
Assistance	Discount Card Program
Assistance	Coupon/Voucher Program
Assistance	Non-Medicare Seniors Card
Unknown	Unknown Third Party
Unknown	Unspecified Plan

© 2023 Strahan AE et al. *JAMA Network Open.*

eTable 2. Product Name Among Retail Pharmacy–Dispensed Buprenorphine Prescriptions by Payer Type, 2020

	**Primary Insurance Payer**					
**Product Name, n (%)**	**Private/ Commercial**	**Self-Pay**	**Medicaid**	**Medicare**	**Assistance**	**Unknown**
**Bunavail**	2,265 (0.08)	287 (0.03)	2,346 (0.04)	417 (0.03)	1,829 (011)	598 (0.06)
**Buprenorphine**	8 (0.00)	2 (0.00)	4 (0.00)	17 (0.00)	7 (0.00)	0 (0.00)
**Buprenorphine Hydrochloride**	442,707 (15.96)	407,746 (47.30)	439,221 (7.57)	190,668 (13.91)	378,978 (23.08)	136,000 (13.10)
**Buprenorphine-Naloxone**	2,003,220 (72.20)	409,524 (47.51)	2,917,650 (50.30)	858,517 (62.62)	1,081,024 (65.85)	763,810 (73.59)
**Sublocade**	22 (0.00)	0 (0.00)	188 (0.00)	17 (0.00)	0 (0.00)	6 (0.00)
**Suboxone**	206,645 (7.45)	40,645 (4.72)	2,339,136 (40.33)	269,210 (19.64)	145,637 (8.87)	82,084 (7.91)
**Subutex**	0 (0.00)	2 (0.00)	0 (0.00)	0 (0.00)	0 (0.00)	0 (0.00)
**Zubsolv**	119,409 (4.30)	3,715 (0.43)	101,204 (1.74)	51,880 (3.78)	34,235 (2.09)	55,329 (5.33)
**Missing**	120 (0.00)	56 (0.01)	186 (0.00)	175 (0.01)	1 (0.00)	75 (0.01)
**Total, n**	2,774,396	861,977	5,799,935	1,370,901	1,641,711	1,037,902

Notes: Authors' analysis of IQVIA Longitudinal Prescription (LRx) database from 2015-2020. Sample includes buprenorphine MOUD prescriptions to adults (aged ≥18 years old) in the United States. Buprenorphine formulations (Butrans and Belbuca) primarily used to treat pain were excluded. All percentages are column percentages. Private/commercial included several forms of employer-sponsored health insurance, plans purchased through health insurance exchanges, and those administered by pharmacy benefit managers. Self-pay indicates a prescription was paid for entirely with cash. Medicaid includes Medicaid managed care or fee-for-service Medicaid. Medicare indicates prescriptions paid by Medicare Part D. The assistance category indicates payment using a discount card (including non-Medicare senior discount cards), a coupon, or a voucher. The unknown category was comprised of prescriptions that were missing payer type or had "unspecified third party" or "unknown" for payer type.

© 2023 Strahan AE et al. *JAMA Network Open.*
