# Supplementary material for: Trends in Out-of-Pocket Costs for and Characteristics of Pharmacy-Dispensed Buprenorphine Medications for Opioid Use Disorder Treatment by Type of Payer, 2015 to 2020
Source: JAMA Netw Open. 2023 Feb 10;6(2):e2254590. doi: 10.1001/jamanetworkopen.2022.54590 (PMC9918874; doi:10.1001/jamanetworkopen.2022.54590)
Supplement: Supplement 2. — Data Sharing Statement [file jamanetwopen-e2254590-s002.pdf]

Data Sharing Statement

Strahan. Trends in Out-of-Pocket Costs for and Characteristics of Pharmacy-Dispensed Buprenorphine Medications for Opioid Use Disorder Treatment by Type of Payer, 2015 to 2020. *JAMA Netw Open*. Published February 10, 2023. doi:10.1001/jamanetworkopen.2022.54590

Data
Data available: No

Additional Information
Explanation for why data not available: The original data used in this analysis were obtained from IQVIA. IQVIA has restrictions prohibiting the authors from making the data publicly available. Interested researchers may contact IQVIA to gain access to the data.
